# Learning behavior, digital platforms for learning and its impact on university student’s motivations and knowledge development

**DOI:** 10.3389/fpsyg.2022.933974

**Published:** 2022-11-23

**Authors:** Uzma Noor, Muhammad Younas, Hessah Saleh Aldayel, Rashid Menhas, Xu Qingyu

**Affiliations:** ^1^School of Education, Soochow University, Suzhou, China; ^2^Department of English Language Skills, King Saud University, Riyadh, Saudi Arabia; ^3^Research Center of Sport and Social Sciences, Soochow University, Suzhou, China

**Keywords:** learning behavior, digital platforms, technological applications, student motivation, knowledge development

## Abstract

**Background:**

Learning digital technologies in higher education is a process of knowledge generation, and the rapid growth of technology in education has a significant impact on students’ learning behaviors, motivation, and knowledge development. Pakistan’s remarkable technological breakthrough has increased in the education field.

**Study objectives:**

The study focuses on estimating students’ learning behaviors, identifying the positive influence of educational apps on digital learning platforms, and analyzing their impact on students’ motivation and knowledge development.

**Materials and methods:**

According to the study’s objectives, a questionnaire survey was conducted to gather the primary data. The participants were students of universities in Lahore city of Pakistan. For this study, the sample size was *N* = 300, carefully chosen using the purposive sampling technique. Of the respondents, there were 146 male and 154 female students, and the sample consisted of individuals aged 25–35 years. Smart-PLS-Bootstrapping, T-Values (PLS) 3.2.9 and the structural equation model (SEM) were applied to get the appropriate outcomes from the proposed study framework.

**Results:**

SEM analysis results shows that all proposed hypotheses [Animated Movies (AM) –> Student Motivation (SM), Educational Apps (EA) –> Knowledge Development (KD), Learning Behavior (LB) –> Animated Movies, Learning Behavior –> Educational Apps, Learning Behavior –> Knowledge Development, Learning Behavior –> Virtual Classrooms (VCr), Virtual Classrooms –> Knowledge Development, Virtual Classrooms –> Student Motivation] are confirmed while Learning Behavior –> Student Motivation is not confirmed.

**Conclusion:**

This study found that digital learning platforms significantly impact students’ learning and what motivates them to learn. The study also found that using educational apps and virtual classrooms more often helps students learn more and be more motivated to learn.

## Introduction

This study examines the use of digital technology in higher education and the learning process of knowledge creation. Web technology is becoming more critical in many areas of Pakistan, including education. The rapid rise of Web technology in teaching and its effect on how people learn encourages students to use technology in the classroom. Interactive teaching tools may enhance the students’ active learning habits. The polytechnic university of the Philippines surveyed students’ attitudes about online and remote learning, motivation, and learning practices (Avila et al., 2021; Shieh and Hsieh, 2021). Recent studies compare direct lectures, interaction, and self-efficacy as determinants of online motivation and satisfaction in the COVID-19 pandemic scenario (Rahman et al., 2021). The research explores students’ learning behaviors and academic achievement in a comparative examination of certain faculty strata and gender and the impact of technological applications, objectives, and time flexibility on students’ digital learning behaviors (Farid et al., 2014; Zhang, 2021). The researchers looked at what factors influenced student learning online during the COVID-19 pandemic to conclude that all educational institutions, teachers, and students need to embrace technology and hone their digital literacy to keep up with the latest global trends and realities in education (Qiuhan et al., 2020; Younas et al., 2022a).

Web conferencing in education helps both educators and students achieve their critical goals of creating the ideal learning environment for kids and balancing life and teaching tasks for educators. Following the definition of online learning, an overview of recent pedagogical strategies has been used in online learning environments (Hartnett, 2016). It is important to note that students’ self-efficacy in computer and online learning, perceived utility, and simplicity of use are essential success criteria in online learning environments (Barclay et al., 2018). The critical challenges for current information technology integrated education are creating educational activities for digital learning and deploying technology tools flexibly. Higher education institutions may use web conferencing to improve instructors’ online access and create new teaching and learning opportunities (Lin and Chen, 2017; Adipat, 2021). The research evaluates the usefulness of e-learning technologies in teaching public institutions in the United Arab Emirates, focusing on course administration and interaction with the students’ information systems (Sandybayev, 2020). Passey and Higgins (2011), Sousa and Rocha (2018) Studies focus on digital learning and motivation among higher education students using an open education platform. These studies focus on current insights on learning outcomes resulting from the usage of learning platforms in schools by learners, students, and instructors. Younas et al. (2022b) indicate how homeschooled English language learners feel about different homeschooling platforms and apps, formulate learning principles, and analyze the broader implications of real-world learning. Vorwerk and Engenhart-Cabillic (2022), Long et al. (2021) discuss how students felt about a newly established digital instructional program, which included an interactive e-book and short learning videos on a YouTube channel. It looks at the connection between how people learn and how well they do in a course, and the flaws in the blended learning mode, which is a big help in improving the quality of education. Finally, the essential components of excellent education are digital platforms for learning and their influence on student motivation and knowledge growth. Digital platforms for learning have unique properties that assist the long-term development of education. The present study explores the learning behaviors and how digital learning impacts students’ motivation and study circles among confident objectives: (1) to estimate the learning behavior of students; (2) to identify the positive influence of educational apps on digital learning platforms; and (3) to analyze the impact of digital learning on students’ motivation and knowledge development.

### Statement of the study

**FIGURE 1:**
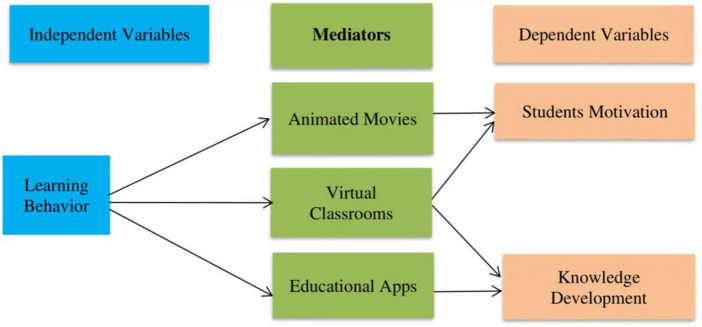
The relationship between different variables of the study.

This study aimed to determine the attractiveness of digital learning platforms and their impact on learning behaviors and knowledge development. The study illustrates that using interactive components of e-learning boosts the motivation of undergraduate students for the learning process (Abou El-Seoud et al., 2014). The research examines students’ self-efficacy, attitudes, confidence in utilizing technology, instructional tactics, the capacity to monitor and assess educational results, and student motivations (Hongsuchon et al., 2022). Game-based learning outcomes were examined, including academic success, problem-solving, critical thinking, and student attitudes and behaviors (Yu et al., 2021). The study looks at the self-regulated learning (SRL) techniques that students use in massive open online courses, emphasizing how learners’ reasons for participating impact their behavior and use of SRL tactics (Littlejohn et al., 2016). According to the research, the social regulation-based online learning strategy improves students’ learning outcomes and motivation (Hwang et al., 2021). As a result, the study indicated that widespread usage of educational apps and virtual classrooms provides better performance in motivating students and knowledge development. The proposed relationships of study variables have been shown in Figure 1, and the hypotheses are given below.

## Literature review

### Digital platforms influence on improving students’ learning behavior

Lai et al. (2016) examined an online training platform that offered pedagogical rationales for self-directed learning and a strategic foundation for aligning technology choices with learning objectives and processes. According to Faridah et al. (2020), the objective of the research is to quantify students’ interest in and engagement with digital learning and investigate how students’ interest in and engagement with digital learning changed throughout the COVID-19 epidemic. Singh et al. (2021) attempt to explain the epochal correlations between the constructs and examine how information might be utilized to increase student acceptance of digital collaboration platforms. Aduba and Mayowa-Adebara’s (2022) study examines how internet platforms were used during the lockdown and (Luo et al., 2019) interpersonal connections have a good influence on the learners’ experience, strengthening their desire to utilize the e-learning platform. Cui et al. (2022) highlight the benefits of combining autonomy with structural assistance and the importance of instructors in a web-based inquiry learning environment. Kang and Zhang (2020) suggest that online education is becoming more popular globally because it engages and motivates students. Panigrahi et al. (2018) reported that technology is being used around the globe to improve education, minimizing the time and space issues associated with conventional learning. The survey results of Moroccan EFL university students are presented on the influence of digital technology on learning behavior and reading motivation. Most students use digital resources for learning and entertainment (Larhmaid et al., 2019).

### Digital platforms’ impact on students’ motivation

Learning motivation is critical for success in online learning, particularly for K-12 students, and the impacts of K-12 students’ perceived presence and technical acceptability in online learning (Zuo et al., 2021). Abou El-Seoud et al. (2014) research shows that interactive e-learning features increase undergraduate students’ interest in learning. Sandybayev (2020) examined how digital technology may help culturally diverse students succeed in school by interviewing 46 students from different academic programs. Flipped teaching may encourage underachieving students to study acting and improve their learning efficacy and psychological needs through self-determination theory. (Chou et al., 2021; Chiu, 2022) studies examined student engagement in online learning and its effectson students learning outcomes. The study aims to determine if there is a strong correlation between e-learning and student motivation in higher education and the increasing use of e-learning (Harandi, 2015). It is more important than ever to figure out how to create electronic learning resources that cater to student motivation and make learning easier (Song and Bonk, 2016), and the study’s goal was to see how the design of a virtual learning environment affects adult learners’ motivation at work (Bashshar, 2017).

### Digital platforms’ impact on students’ knowledge development

Recent studies show that the quick shift from traditional to digital learning has harmed students’ intrinsic and extrinsic motivation (Gustiani, 2020). Education-related departments strive to positively cultivate students’ professional information knowledge and skills (Sun and Pan, 2021). Rippa and Secundo’s (2019) work adds to the burgeoning notion of digital academic entrepreneurship, and Díaz-Noguera et al.’s (2022) study gives a development model of students’ adaptive capabilities to the digital revolution in university education. The study finds the essential abilities and information from studies that reinforce each other and help people learn more deeply (Makani et al., 2016). This research examined how tourism and hospitality students see how sharing economic platforms help them learn and develop their beliefs and attitudes (Horng et al., 2022). According to the findings, which looked at students’ motivation for online learning, most students said they were engaged in class and had strong time management skills while taking online courses (Cabansag et al., 2020).

### Theoretical framework

Social cognitive theory (SCT) stresses learning from the social environment (Schunk and Usher, 2012). A key feature of SCT is its emphasis on external and internal social reinforcement. In the social climate, SCT stresses the unique way humans learn and maintain behavior. These earlier experiences build attitudes and expectancies, which decide whether or not a person engages in a particular activity. People engage in diverse vicarious, symbolic, and self-regulatory processes to build a feeling of agency. In addition to objectives and self-evaluations of achievement, key motivators include values, peer comparisons, and self-efficacy. People establish and track objectives. Progress perception boosts self-efficacy and motivation. People operate according to their principles and seek the desired results. Other people’s learning and goal achievement might be compared. Self-efficacy influences task choices, effort, perseverance, and accomplishment. Depending on their social surroundings, humans might be proactive, engaged, or estranged (Ryan and Deci, 2000). To this end, social–contextual elements that encourage or impede self-motivation and healthy psychological development have been studied. Specific variables, such as enhancing or reducing intrinsic motivation, self-regulation, and wellbeing were studied.

### Hypotheses of the study

**H1:** Learning behavior (LB) is positively associated with watching animated movies (AMs).

**H2:** Student’s motivation (SM) is positively influenced by AM and LB.

**H3:** LB is positively impacted SM through virtual classroom (VCr).

**H4:** LB positively influences the use of educational apps (EAs).

**H5:** Knowledge development (KD) is positively influenced by VCr and LB.

**H6:** KD is positively influenced by the use of EAs and LB.

## Research method

### Study locale and population

The present study has been conducted in Lahore, the capital of Punjab Province in Pakistan and is exploratory. The online survey was used to gather primary data according to the research goals. The research goal was explained to all survey participants and their agreement was obtained. The researchers did a quality check while the data were being gathered. All the people who participated in the study were entirely voluntary, and they were told their information would only be used for research purposes.

### Sampling

The sample size for this research was *N* = 300, which was chosen using purposive sampling. The criteria mentioned were students with four majors (such as social sciences, psychology, management studies, and education) who participated in this study and had different educational backgrounds. Finally, 75 students were selected from each primary subject, and participants of different genders and age groups participated in this study (Liébana-Presa et al., 2020). The information gathered during data collection was divided into categories depending on the frequency and percentages of each question in demographics, and Table 1 summarizes the findings. There were 146 male and 154 female students among the responders. Eighty-three respondents were under 25 years, 129 were between the ages of 25 and 30 years, and 88 were between the ages of 31 and 35 years. Similarly, for the question, 29 respondents belonged with a doctoral degree, 107 were from master’s degree, 95 were from bachelor’s degree, and 69 respondents had an associate degree. Similarly, students with four different significant studies (such as social sciences, psychology, management studies, and education) participated in this study, and 75 male and female students were chosen from each major equally to get unbiased data (please see Table 1).

**TABLE 1:** Demographic information of participants.

Demographic summary	Category	Frequency/Percentage
Gender	Male	146(48.66%)
	Female	154(51.33%)
Age	20–25	83(27.66%)
	25–30	129(43%)
	30–35	88(29.33%)
Qualifications	Doctoral	29(9.66%)
	Master	107(35.67%)
	Bachelor	95(31.67%)
	Associate	69(23%)
Study fields	Social sciences	75(25%)
	Psychology	75(25%)
	Management	75(25%)
	Education	75(25%)

### Data collection process

The survey’s approach was employed to gather data, and a questionnaire on learning behavior and the influence of students’ motivation and knowledge growth was created. It was built on a five-point Likert scale, with one indicating strong agreement and five indicating extreme disagreement, and the scale was modified based on previous studies (DeVries et al., 2018). Dependent on the availability of participants, questionnaires were used to gather data, and the data for this research were gathered at Pakistani universities. Respondents filled out these surveys, and the demographic employed in this research included university students attending courses on learning practices, digital platforms, and their effects on knowledge acquisition. A survey was performed to gather primary data according to the research goals. The research goal was explained to all survey participants and their agreement was obtained. The researchers did a quality check while the data were being gathered. The survey participants’ anonymity and the confidentiality of the received information were guaranteed. While getting informed consent, it was clear that all data would only be used for research.

### Operationalization of study variables

The questionnaires included six variables to gather data, and 23 items were included in the questionnaires. The study’s conceptual framework contained one independent variable (such as learning behavior), three mediators (namely animated movies, virtual classrooms, and educational Apps), and two dependent variables (such as student motivation and knowledge development). Some previous studies contain one independent variable, three or four moderators, and one or two dependent variables (Lin and Chen, 2017; DeVries et al., 2018).

### Demographic information

Considering the previous research (Salkind, 2010), the study contains gender, age, qualifications, and study fields as demographic factors. All self-reported variables were categorized by gender (male or female), age (between 20 and 35 years), qualification (namely doctoral, master, associate, and bachelor), and study major (such as social sciences, psychology, management studies, and education).

### Digital learning impact

Learner motivation and learning outcomes are better in digital education than in traditional education. Motivating oneself to learn has a significant favorable impact on one’s learning ability. To determine the elements impacting students’ happiness and performance in online courses and their interaction (Gopal et al., 2021). The digital learning impact on students was assessed by using the Likert-scale questions about AM, VCRs, and EAs.

### Students’ motivation and knowledge development

Student motivation plays a pivotal role in knowledge development during learning with technological aspects. Developing student motivation is a challenging but vital part of teaching that instructors must address. This research aimed to see how technology affects students’ drive to learn and remember new material (Granito and Chernobilsky, 2012; Yarborough and Fedesco, 2020). The survey participants were assessed through motivation and knowledge development questions.

## Results

Smart PLS-Bootstrapping, T-Values (PLS) 3.0 and structural equation modeling (SEM) were used to examine the model, including internal consistency reliability, convergent validity, and discriminant validity as examples of indicator loadings (Hair et al., 2019). With the help of SEM, the Smart-PLS study strategy is a robust, scalable, and cutting-edge approach to creating a substantial statistical model (Abbas et al., 2019a). PLS-SEM looks at complicated models with both observable and latent parts. It may be able to give SEM results with different levels of structural complexity, such as higher-order structures that often solve problems with multicollinearity and look into the measurement and structural models (Ringle et al., 2015; Sharif et al., 2021).

### Internal consistency reliability

The Internal consistency reliability (ICR) was implemented to assess the consistency of findings across indicators. The present technique reported Cronbach’s alpha and composite reliability (CR). ICR values should range from 0 to 1. Cronbach’s alpha and Cronbach’s coefficient of determination (CR) should be more than 0.700. Cronbach’s alpha and Cronbach’s CR reports are shown in Table 2. All constructs have a good Cronbach’s alpha, and their CR values meet or exceed what is needed.

**TABLE 2:** Reflective indicator loadings, internal consistency reliability, and convergent validity.

Constructs	Items	Loadings	VIF	Alpha	CR	AVE
Animated movies	AMs_1	0.898	1.956	0.726	0.846	0.650
	AMs_2	0.855	1.773	–	–	–
	AMs_3	0.644	1.230	–	–	–
Educational apps	EAs_1	0.723	1.076	0.767	0.734	0.580
	EAs_2	0.607	1.098	–	–	–
	EAs_3	0.742	1.105	–	–	–
Knowledge development	KD_1	0.799	1.460	0.774	0.869	0.689
	KD_2	0.845	1.722	–	–	–
	KD_3	0.846	1.680	–	–	–
Learning behavior	LB_1	0.533	1.143	0.714	0.755	0.587
	LB_2	0.562	1.236	–	–	–
	LB_3	0.536	1.182	–	–	–
	LB_4	0.803	1.280	–	–	–
	LB_5	0.635	1.285	–	–	–
Students motivation	SM_1	0.752	1.701	0.869	0.906	0.659
	SM_2	0.872	2.808	–	–	–
	SM_3	0.739	1.826	–	–	–
	SM_4	0.806	1.953	–	–	–
	SM_5	0.878	2.700	–	–	–
Virtual classrooms	VCr_1	0.861	2.046	0.791	0.864	0.621
	VCr_2	0.849	1.952	–	–	–
	VCr_3	0.854	1.914	–	–	–
	VCr _4	0.541	1.210	–	–	–

AMs, animated movies; EAs, educational apps; KD, knowledge development; LB, learning behavior; SM, student motivation; VCr, virtual classrooms.

Animated movies had a Cronbach’s alpha of 0.726 and a CR of 0.846, while educational apps had an alpha of 0.767 and a CR of 0.734. Knowledge development possessed an alpha of 0.774 and a CR of 0.869. Learning behavior had an Alpha of 0.714 and a CR of 0.755. Students’ motivation possessed an alpha of 0.869 and a CR of 0.906. Finally, virtual classrooms obtained an alpha of 0.791 and a CR of 0.864.

### Variance inflation factor

The prediction skills of the structural model were tested as part of the evaluation. However, the collinearity value should be indicated before providing the structural model by reporting the variance inflation factor (VIF) values. Notably, the predictors/mediators were assessed for the collinearity of animated movies, educational apps, and virtual classrooms as mediators of learning behavior, student motivation, and knowledge development, respectively (Hair et al., 2019). VIF levels should be less than three; values greater than three are generally associated with multicollinearity issues. According to the data analysis, all VIFs are less than three. As a result, collinearity is not a concern in this study’s model.

### Convergent validity

Convergent validity is a subtopic of construct validity in which tests with the same or comparable constructs should be substantially connected. The average variance derived from this research is used to calculate the convergent validity average variance extracted (AVE). The AVE was calculated using Smart PLS 3.0. According to the methodology, AVE values should be 0.500 or higher, explaining 50% or more of the variation (Table 2). All constructs had AVE values of more than 0.500, indicating that they presented more than half of the variation. Animated movies’ AVE value was 0.650, educational apps’ AVE value was 0.580, knowledge development’s learning behavior was 0.587, student motivation’s AVE value was 0.659, and virtual classrooms were 0.621.

### Loading indicators

**FIGURE 2:**
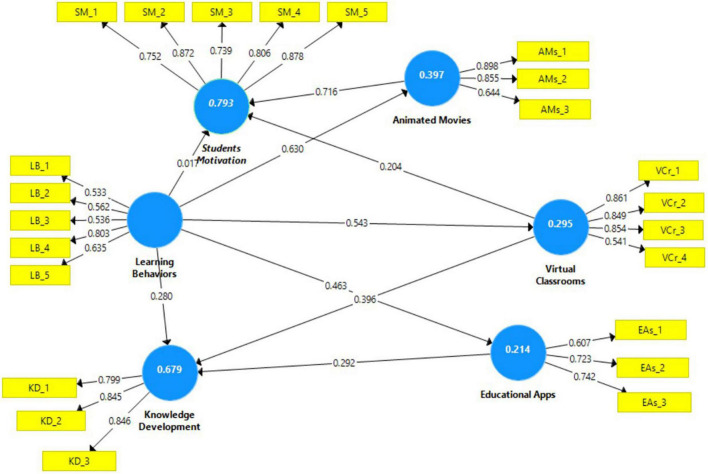
PLS-structural equation modeling (SEM) results.

Figure 2 shows the factor loadings acquired by PLS-SEM to confirm the validity. The loadings of reflective indicators attained in SEM should be more than 0.500, and all loadings should be greater than 0.500 based on the calculation. “Animated Movies (0.898, 0.855, 0.644), Educational Apps (0.723, 0.607, 0.742), Knowledge Development (0.799, 0.845, 0.846), Learning Behavior (0.533, 0.562, 0.536, 0.803, 0.635), Students Motivation (0.752, 0.872, 0.739, 0.806, 0.876), and Virtual Classrooms (0.861, 0.849, 0.854, 0.541).”

### Discriminant validity

Discriminant validity (DV) showed how to quantify constructs that were conceptually unrelated to one another. Discriminant validation seeks to show any discriminating evidence based on all components’ dissimilarities (Campbell and Fiske, 1959). The overlap of measurements on each other is used to assess discriminant validity (please see Table 3). Comparing the square root of a factor’s AVE values with the correlation between constructs might indicate DV. AVE values should be greater than correlations (Campbell and Fiske, 1959). Table 2 shows that the AVE square root is more significant than correlation values, which indicates good assessment.

**TABLE 3:** Discriminant validity (*N* = 300).

	AMs	EAs	KD	LB	SM	VCr
Animated movies	0.806	–	–	–	–	–
Educational apps	0.669	0.693	–	–	–	–
Knowledge development	0.705	0.698	0.830	–	–	–
Learning behaviors	0.630	0.463	0.631	0.622	–	–
Students motivation	0.880	0.737	0.743	0.579	0.812	–
Virtual classrooms	0.754	0.698	0.752	0.543	0.753	0.788

AMs, animated movies; EAs, educational apps; KD, knowledge development; LB, learning behavior; SM, student motivation; VCr, virtual classroom.

### Model fit summary

This work’s model fitness was assessed using Standardized-root-mean-square-residual (SRMR), normed fit index (NFI), and Chi-square (X^2^). It is a measure of model fitness that compares observed covariance to hypothesized matrices (Chen, 2007; Brown, 2015). The SRMR value must be less than or equal to 0.08 to be considered acceptable. Results show that the predicted SRMR value of 0.079 is a satisfactory model fit for the standardized root mean square residual. An NFI score of 0.475 and a X^2^ value of 2868.490 (please see Table 4) indicate that the two datasets are statistically insignificant.

**TABLE 4:** Model fit summary.

Statistical tests	
SRMR	0.079
d_ULS	8.380
d_G	2.319
X^2^	2868.490
NFI	0.475

SRMR, standardized-root-mean-square-residual; d_ULS, unweighted least squares discrepancy, d_G, geodesic discrepancy; X^2^, chi-square, NFI, normed fit index.

### PLS-bootstrapping, T-values

**FIGURE 3:**
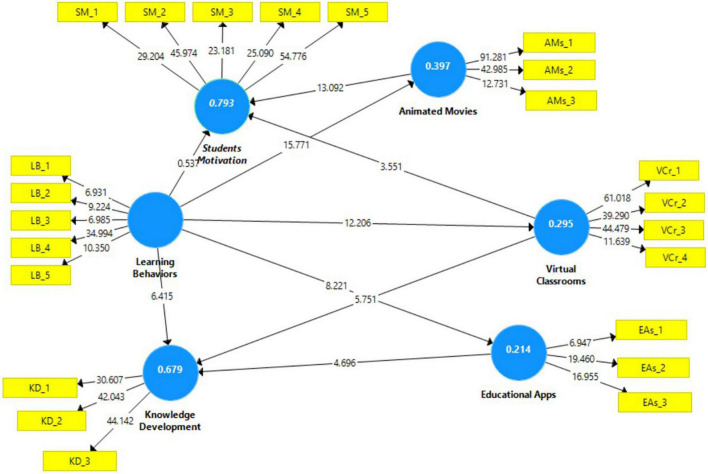
PLS bootstraping.

The import of all straight effects was assessed for the structural model by examining the path coefficients, T-statistics, and *p*-value. We computed the data through a bootstrapping procedure. The bootstrapping computation results are presented in Table and Figure, with the Table informing the hypotheses, relationship, path, T-value, and *p*-value. Figure 3 illustrates the T-value and loading value of the path lines during the bootstrapping process.

The hypotheses’ statistical significance was assessed using a standard beta calculation. We can see how much the dependent component may vary from the independent factor using the beta number. Each association’s standardized beta (β) value was determined following the predicted study model (Table 5). High and significant beta (β) values indicate that endogenous latent variables have a strong influence. T-statistics were utilized to validate the significance of the beta value for each route in the experiment. The significance level of putative associations was assessed and evaluated using the beta (β) value acquired using the bootstrapping approach. The structural model’s hypothesized connections are shown in all beta (β) values in Figure 3, Table 5, respectively, to illustrate the link between T-values and observed variables (see Figure 3).

**TABLE 5:** Path, T-value, and *P*-value.

H	Relationships	Path (β)	T-value	*P*-value	Decision
H1	AMs –> SM	0.716	13.092	0.001	Confirmed
H2	EAs –> KD	0.292	4.696	0.001	Confirmed
H3	LB –> Ams	0.63	15.771	0.001	Confirmed
H4	LB –> Eas	0.463	8.221	0.001	Confirmed
H5	LB –> KD	0.28	6.415	0.001	Confirmed
H6	LB –> SM	0.017	0.537	0.592	Not-Confirmed
H7	LB –> VCr	0.543	12.206	0.001	Confirmed
H8	VCr –> KD	0.396	5.751	0.001	Confirmed
H9	VCs –> SM	0.204	3.551	0.001	Confirmed

AMs, animated movies; EAs, educational apps; KD, knowledge development; LB, learning behavior, SM, student motivation, VCr, virtual classrooms.

The maximum T-value was attained by the path between facilities and research activities (*t* = 15.771), while the last value was the association between practical activities and employability (*t* = 0.537). All hypotheses projected in this study were supported. In detail, H1 was reported to be significant in influencing students’ motivation (β = 0.716; *t* = 13.092; *p* < *0.001*) and knowledge development. H2 reveals that educational apps have a significant impact (β = 0.292; *t* = 4.696; *p* < 0.001) on knowledge development. H3 was also supported where learning behavior is significantly predicted by animated movies (β = 0.630; *t* = 15.771; *p* < 0.001). Similarly, the significant role of learning behavior in educational apps (H4) was also reported (β = 0.463; *t* = 8.221; *p* < 0.001). Learning behavior is also a significant predictor for knowledge development, H5 (β = 0.280; *t* = 6.415; *p* < 0.001). The result of PLS-SEM supports H6 because there is a direct effect of learning behavior on student motivation (β = 0.017; *t* = 0.537; *p* < 0.592). H7 is also supported as learning behavior is significantly predicted by virtual classrooms (β = 0.543; *t* = 12.206; *p* < 0.001). Finally, the findings also support hypotheses 8 and 9. Positive relationships also emerged between virtual classrooms and knowledge development (β = 0.396; *t* = 5.751; *p* < 0.001). Virtual classrooms are also known to be a significant predictor of students’ motivation (β = 0.204; *t* = 3.551; *p* < *0.001*).

## Discussion

It was determined that e-learning was successful and investigated major antecedents of e-learning success in the COVID-19 pandemic (Elshaer and Sobaih, 2022; Jaoua et al., 2022). The major motivators for the likely continuance of videoconferencing as a supplement to face-to-face tutorials at Spanish institutions (Infante-Moro et al., 2021). Sequential analysis is used to assess the impact of student motivation on online reading behavior and the perceived online social presence in an online course (Tao, 2009; Sun et al., 2018). According to the result of the SEM model, these hypotheses (H1: Animated Movies –> Student Motivation, β = 0.716; *t* = 13.092; *p* < 0.001*;* H2: Educational Apps –> Knowledge Development, β = 0.292; *t* = 4.696; *p* < 0.001; H3: Learning Behavior –> Animated Movies, β = 0.630; *t* = 15.771; *p* < 0.001; H4: Learning Behavior –> Educational Apps, β = 0.463; *t* = 8.221; *p* < 0.001; H5: Learning Behavior –> Knowledge Development, β = 0.280; *t* = 6.415; *p* < 0.001) are confirmed. The result of H6 indicates that (Learning Behavior –> Student Motivation, β = 0.017; *t* = 0.537; *p* < 0.592) there is no direct effect of learning behavior on student motivation. The approach suggests three characteristics to enhance students’ ability to adapt to digital change in university teaching: motives, digital pedagogy, and student autonomy (Hwang et al., 2015; Díaz-Noguera et al., 2022). Since information and computer technology (ICT) skills are becoming more important everywhere, especially in the workplace, one of the main goals of colleges has been to teach students how to deal with problems (Bond et al., 2018; Muhammad et al., 2020). Previous research examines the effect of enhancing overall wellness during the COVID-19 epidemic and university students’ dangerous online activity (Peng et al., 2022).

In education, digital transformation is a long-term process that has become a pressing issue. Studies indicate a positive association between openness to experience and creativity and a mediation function for intrinsic drive and creative process participation in this relationship (Tan et al., 2019; Bogdandy et al., 2020). The study analyzed teacher opinions on how technology affects student academic behavior and performance in a blended learning environment and also looked at students’ behavioral intentions (motivation) for adopting online learning technologies (Chen et al., 2002; McHone, 2020). The analysis results show that these hypotheses (H7: Learning Behavior –> Virtual Classrooms, β = 0.543; *t* = 12.206; *p* < 0.001: H8: Virtual Classrooms –> Knowledge Development, β = 0.396; *t* = 5.751; *p* < 0.001; H9: Virtual Classrooms –> Student Motivation, β = 0.204; *t* = 3.551; *p* < 0.001) are confirmed. To construct an efficient 21st century classroom that fits the requirements of students, a modern teacher must consider a student’s drive to study and the effect of technology has on inclusionary education (Granito and Chernobilsky, 2012; Kizilcec and Schneider, 2015). There has never been a study that looked specifically at how university students’ learning attitudes are affected by social media’s good and bad features (Abbas et al., 2019b).

Online education is rapidly developing due to a growing demand for higher and continuing education. Yet, many online students fail to fulfill their educational objectives due to the lack of face-to-face interaction (Zheng et al., 2015; Kizilcec et al., 2020). Learning behavior is also a significant predictor of knowledge development in H5 (β = 0.280; *t* = 6.415; *p* = 0.000). H6 results show that there is a direct effect of learning behavior on student motivation (β = 0.017; *t* = 0.537; *p* = 0.592). Students’ willingness to attend online courses was predicted using the Motivation Orientation Scale and the Unified Theory of User Acceptance of Technology (Cullum, 2016). The capacity of students to persevere and get high marks when taking online classes is compared with their ability to do so while taking face-to-face courses (Xu and Jaggars, 2013). Investigations were carried out to determine how strong the link between online learning and students’ motivation was among the participants and whether there was a direct correlation between student views of their motivation to read and their accomplishments in a blended learning environment (Minda, 2020; Miller, 2021). Researchers wanted to determine how e-learning activities interacted with such characteristics as gender, maturity level, field of study, geographic location, and grade level (Thomas, 2020). As the world becomes more digital, it is important to define information literacy and investigate studies on game-based learning (Press, 2017). This research found that digital platforms significantly influence learning behavior and affect students’ motivation and knowledge growth. Learner motivation and learning outcomes are greater in digital education than in traditional education.

## Conclusion

With the growing usage of digital platforms for learning, the introduction of various technology applications, and their influence on learning behavior, student motivation, and knowledge acquisition, the whole domain of learning and education in Pakistan and throughout the globe has altered. This study also examines students’ studying habits in light of digital learning’s impact on education. Intentions for digital learning are more vivid in students who participate in digital learning. Motivating oneself to study has a substantial favorable effect on one’s learning ability. Furthermore, goal-setting behavior and social pressures have increased students’ digital learning practices. These results have been a significant step forward in creating knowledge and improving student learning. The findings of this study have a wide range of ramifications for future academics and organizations that want to replicate this research in various places with their resources. New research paths like these may benefit significantly from the use of these.

### Study limitations

There are several limitations regarding the current study. The respondent must be 20 + years old university student. There are a lot of digital platforms for academic learning. Some of them are freely available and some of them are paid or subscription-based. Most universities have paid or subscription-based access to their students’ digital platforms of academic learning. Only those university students can participate in the study survey that uses their universities’ available digital platforms. Purposive sampling was used as part of the non-probability sampling (NPS) technique since it best suited the study’s goals and objectives. The conclusions of this research cannot be extended to the whole population since it is difficult to repeat the results of the purposeful sample.

## Data availability statement

The raw data supporting the conclusions of this article will be made available by the authors, without undue reservation.

## Ethics statement

The studies involving human participants were reviewed and approved by School of Education, Soochow University. The patients/participants provided their written informed consent to participate in this study.

## Author contributions

XQ is the principal investigator. UN and MY collected/analyzed data and wrote the manuscript. RM designed the study model and hypothesis and contributed to the discussion section. HS contributed to drafting the literature review, discussion, and overall editing of the manuscript. All authors contributed to the article and approved the submitted version.
